# Integrating ultrastructural diffraction imaging and multiscale modelling to unveil the nanoscale mechanics of arthropod cuticle in bending

**DOI:** 10.1098/rsif.2024.0601

**Published:** 2025-03-19

**Authors:** Yanhong Wang, Ettore Barbieri, Yi Zhang, Nick Terrill, Himadri Shikhar Gupta

**Affiliations:** ^1^ School of Engineering and Materials Science and Institute of Bioengineering, Queen Mary University of London, London, UK; ^2^ Japan Agency for Marine-Earth Science and Technology (JAMSTEC), Research Institute for Value-Added-Information Generation (VAiG), Center for Mathematical Science and Advanced Technology (MAT), 3173-25, Showa-machi, Kanazawa-ku, Yokohama, Japan; ^3^ Diamond Light Source, Harwell Science and Innovation Campus, Didcot, UK

**Keywords:** chitin fibril, stomatopod cuticle, synchrotron X-ray diffraction, *in situ* realistic multiscale modelling

## Abstract

Determining the mechano-structural relations in biological materials with hierarchical structure is crucial to understanding natural optimization strategies and designing functional bioinspired composites. However, measuring the nanoscale mechanics and dynamic response is challenging when the specimen geometry and loading environment are physiologically complex. To overcome this challenge, we develop a combination of synchrotron X-ray diffraction testing and analytical modelling to explore the mechano-structural changes during bending loads on stomatopod cuticle. Stomatopod cuticle is an example of a hierarchical biomaterial optimized for high impact and bending resistance. Using models for large deformations of elastic continua, we measure cuticle strains from macroscopic deformations and combine diffraction-based fibril strains with stresses to quantify the local elastic moduli and nanoscale strain concentration factors, which are found to vary across cuticle sub-regions and under different flexion loading modes. This approach has the advantage of identifying constituent biomaterial properties and mechanisms *in situ* and is also suitable for studying time-dependent changes, such as concurrent strains of the nanofibrous phase that occur during physiological loading.

## 1. Introduction

**Figure 1.**
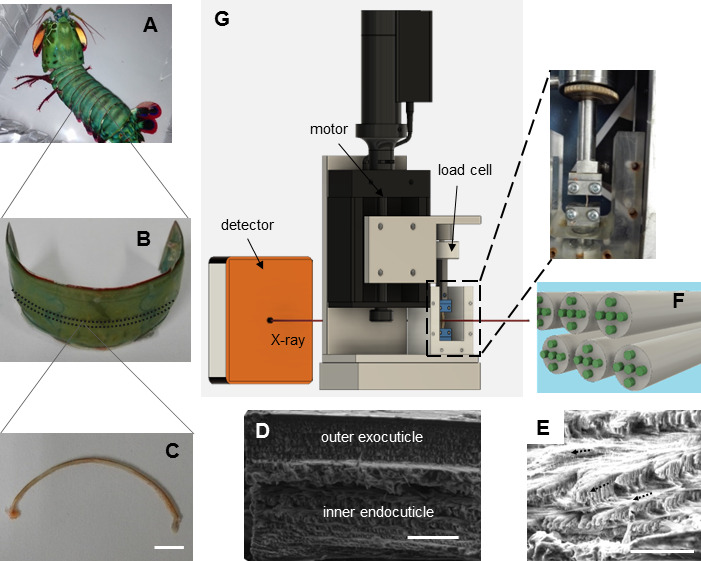
Stomatopod abdominal tergite cuticle structure and experimental set-up for *in situ* mechanics. (A) Picture of a stomatopod. (B) Image of an abdominal tergite after soft residual material was removed. The dash lines indicate one section for the bending test sample. (C) A representative bending test sample (scale bar: 5 mm). Scanning electron microscopy (SEM) images of the fracture surface showing (D) the outer exocuticle and inner endocuticle, and (E) the layers with different orientations (indicated by arrows) in cuticle (scale bars: 100 µm in (D) and (E)). (F) A schematic shows the mineralized chitin-protein fibres (green and grey) embedded in mineral–protein matrix (blue). (G) The micromechanical tester used in line with X-ray beam to simultaneously measure changes in fibril strain while performing the bending test. The inset is a magnified view showing how a sample was mounted on the tester.

Biological armours, such as reptile carapaces, fish scales, seashells and arthropod exoskeletons, play a crucial role in the physiological functioning of these organisms and serve as a source of inspiration for the development of advanced functional materials [1–4]. However, measuring and modelling the mechanical properties at the material level is challenging due to the intricate geometry and hierarchical structure of these natural defences. Stomatopod cuticle provides an excellent system for developing an integrated experimental and modelling approach because it exhibits a multiscale biocomposite structure with a complex hierarchical architecture from nano- to macro-length scales (figure 1) and serves as a prototypical example of structurally graded natural armour [5,6], evolutionarily optimized adaptation to external *in vivo* loads, including impacts from intraspecific competition [7–10].

The stomatopod cuticle comprises three distinct macroscale layers: the epicuticle, which is a thin 1−2 μm waxy surface layer providing waterproofing, and the exocuticle and endocuticle, which are the primary structural and mechanical components [11–15]. The exocuticle and endocuticle consist of chitin fibres wrapped by a protein–mineral matrix. Notably, the external exocuticle exhibits higher biomineralization, including calcium carbonate, calcium phosphate and calcite, resulting in enhanced hardness compared with the less mineralized internal endocuticle [16,17]. At the micro level, the chitin fibres are arranged in a planar plywood (Bouligand) pattern, with a smaller fraction of out-of-plane fibres running perpendicular to the Bouligand layers [18–23]. These multiscale structural characteristics contribute to the excellent impact resistance observed in various arthropod exoskeletons, such as the claws of lobsters and crabs [19,24], and dactyl and telson in mantis shrimp [23,25–28]. Furthermore, these distinctions between regions go beyond composition and structure and show distinct mechanical properties. For example, using indentation-type testing, within the dactyl cuticle, the local modulus of the impact region (approx. 65–70 GPa [23,29]) is much higher than the periodic region or saddle (10–30 GPa) [17], while in the crab cuticle [30], hardness varies by a factor of approximately 2 on going from exocuticle to endocuticle in both claws and legs (e.g. 947 versus 471 MPa in claws). Microscopic or macroscopic tensile tests on cuticle give lower moduli (0.5−0.7 GPa [30]) compared with the typical indentation moduli range above [17,23,29].

Indeed, the mechanical characteristics of stomatopod cuticle are an example of the diverse biological armours found in arthropod and insect cuticle across species, as reviewed recently [3,31]. Considering these as hierarchical materials, factors like hydrogen-bond stabilization and protein/chitin interactions at the fibrillar level are believed to contribute to stiffness. Toughness is enhanced by varying hydration, sclerotization and the cross-ply Bouligand structure. Different mineralization levels also lead to stiffer exocuticle and softer endocuticle. Variation of anisotropic structure and material properties of the cuticle leads to altered stiffness and flexibility across, for example, limbs in the desert locust that have different locomotory roles *in vivo* [32]. Methods to assess the multiscale mechanisms in cuticle include, for example, macroscale impact testing and simulation of fracture in the Bouligand layer [31], anisotropic mechanics using nanoindentation [32] and novel non-contact measures of stiffness using the autofluorescence signal from three-dimensional confocal microscopy, especially appropriate for small cuticular organs with complex shapes [33].

To achieve the desired functional characteristics in cuticle, the mechanics must be adapted at all hierarchical levels, including the smallest molecular to nanometre level of the mineralized fibrils. Nevertheless, the mechanisms at this scale, during loading, remain unclear, especially between and within the chitin fibrils and surrounding (possibly mineralized) protein matrix. Indeed, as noted in [31] (albeit for insect cuticle), there is a gap in our understanding of the interactions at the nanometre level between chitin fibrils and the protein matrix, as well as how these give rise to local as well as macroscopic mechanical properties. We previously used high-brilliance synchrotron X-ray diffraction combined with *in situ* mechanics in simplified loading (tension and compression) to measure fibrillar strain and orientation of the α-chitin microfibrils [6,34,35]. However, the cuticle is a curved shell that is deformed in impact via bending, and that loading mode has not been studied. Bending will involve a mixture of tensile and compressive loading along with shear, and the response may be asymmetric in tension and compression, as is known to occur for other mineralized systems like bone [36]. Due to diffraction geometry and loading constraints coupled with anisotropic and spatially graded geometry, current *in situ* synchrotron diffraction methods face challenges in these realistic loading modes. While recent advances in three-dimensional X-ray diffraction tensor tomographic techniques [37] will enable such methods in the future, here we present proof of concept of an alternative simpler approach—using scanning two-dimensional X-ray diffraction with *in situ* simplified bending, coupled with multiscale analytical modelling. Such a method could also complement larger-scale X-ray tomographic imaging (at the microscale) of cuticle structure [31] and in combination might inform finite-element modelling of intact cuticular organs. It would aid, for example, in separating the contributions of compositional factors (e.g. mineralization and organic composition profile) and structural ones (like fibril pre-strain gradients) on the cuticle mechanical properties.

**Figure 2.**
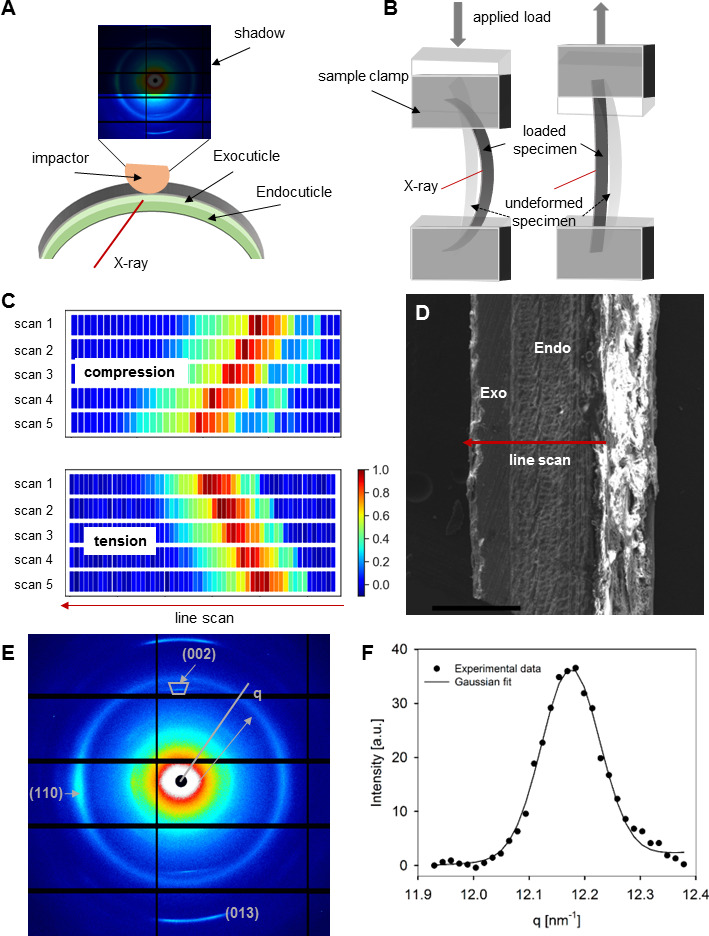
Experimental protocol for *in situ* mechanics analysis of chitin fibrils in cuticle. (A) A schematic shows the impact set-up of an abdominal tergite cuticle, resulting in a shadow in the detector and the X-ray pattern. The light orange object indicates an impactor, such as like a dactyl; the dark red line indicates the direction of the X-ray beam; the light green, the exocuticle; the green, endocuticle. (B) A schematic shows the macroscale deformation of the cuticle during bending test (left: compression, right: tension). The applied load, grey arrow; undeformed and loaded specimens, dashed and solid black arrows, respectively; and X-ray beam, dark red lines. (C) Wide-angle X-ray diffraction (WAXD) intensity mapping of chitin diffractions (the integrated intensity from 11.85 to 19.05 nm^−1^) from individual samples to show the position and the horizontal movement of cuticle during compression (upper) or tension (lower) for multiple X-ray scans at different macroscopic strains of 0% (before loading, scan 1), 0.5% (scan 2), 1.0% (scan 3), 1.5% (scan 4) and 2.0% (scan 5). The arrow line indicates the line scans from endocuticle (right side) to the exocuticle (left side). (D) SEM image of cuticle cross-section area (scale bar: 250 µm) showing the scan position (red line). (E) Representative WAXD pattern showing the diffraction signals of (002), (110) and (013) from α-chitin. (F) One-dimensional intensity profile of diffraction peak (002) with both experimental data (circle) and the Gaussian fit (line).

In summary, our approach aims to extract the nanoscale deformation characteristics *in situ* at the supramolecular level (e.g. Young’s moduli of the matrix in different regions of the cuticle, fibril strain transfer ratio [34]) during such large-scale macroscopic deformations combined with ultrastructural characterization (figure 2). This therefore avoids issues with localized compressive probes on static samples (e.g. nanoindentation), which may not always capture the stress and strain states set up by the spatially extended complex multiaxial deformation modes at the whole organ level. This approach—potentially generalizable beyond cuticle—may shed light on the design principle for bioinspired graded-structural composites.

## 2. Results and model

**Figure 3.**
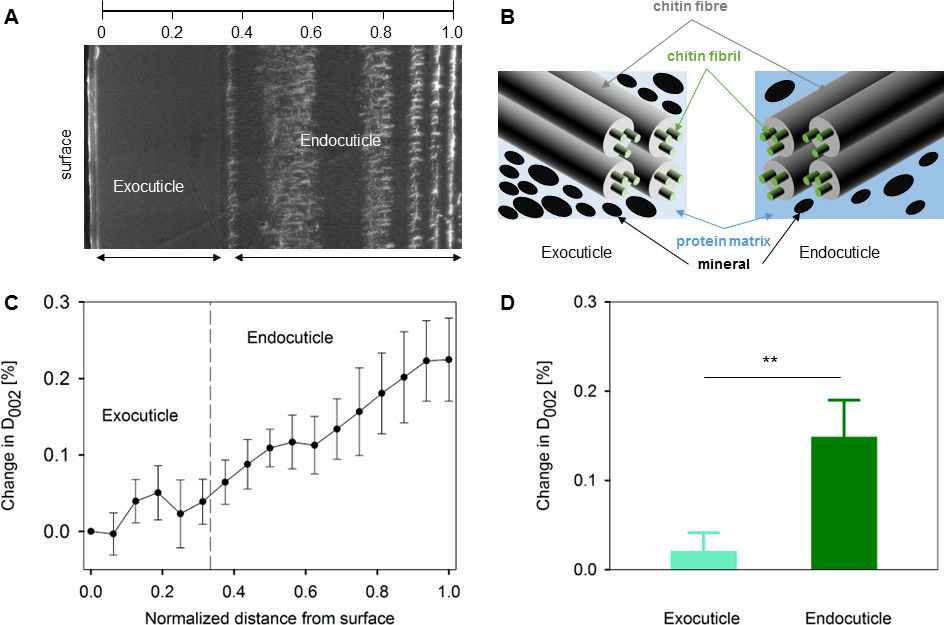
Lattice spacing across cuticle. The change in *D*_(002)_ of chitin fibrils from outer exocuticle to inner endocuticle reveals a continuous gradient through the thickness of the exoskeleton of stomatopod. (A) SEM image of the cross-section showing the structure through the thickness of the cuticle (left side: exocuticle, right side: endocuticle) and the relative position at which the data were taken. The distance is normalized to the thickness. (B) A schematic showing the chitin fibril/fibre, the protein matrix, and the minerals in the two layers—exocuticle and endocuticle. (C) The percentage change in *D*_(002)_ across the thickness of the cuticle. (D) The percentage change in *D*_(002)_ (from the surface) in exocuticle (light green) and endocuticle (dark green). Data is mean ± s.e.m. (s.e.m.: standard error of mean), *n* = 9 in (C) and (D).

As described in §5, stepwise bending experiments on abdominal tergite cuticle were carried out using a customized micromechanical tester, in combination with scanning wide-angle X-ray diffraction (WAXD) to measure the fibril-level strains from shifts in the crystallographic peaks arising from the chitin fibril orthorhombic lattice structure [38,39]. The (002) diffraction peak (which is along the axis of the fibril) is associated with a lattice spacing *D*_(002)_, whose percentage changes are thus taken as a measure of axial fibril strain [6]; the (013) peak will reflect a combination of on-axis and off-axis fibril strains and is hence not used here. The cuticle was thawed in saline, and during the test, a saline-soaked gauze was wrapped on the upper sample holder in contact with the sample to ensure it stayed wet. The experimental configuration is depicted in figure 2. Line scans were taken with a step size of 20 μm across the thickness of the cuticle (figure 3A; encompassing the outer exocuticle and the inner endocuticle) and were laterally offset by 25 μm (X-ray beam diameter 15 μm) between macroscopic strain-steps (§5).

### 2.1. Chitin fibril pre-strain gradient across the cuticle

In the undeformed cuticle, we observed a spatially varying ultrastructural change (figure 3C) in the lattice spacing of the (002) peak of the chitin fibrils *D*_(002)_. The magnitude of *D*_(002)_ increases when going from the exocuticle to the endocuticle, and the percentage change can be calculated from the exocuticle surface. When comparing the percentage change of *D*_(002)_ in exocuticle versus endocuticle, the average change in endocuticle is much larger (by approx. 0.15% pre-strain; *p* < 0.01 between exocuticle and endocuticle; *n* = 9 cuticle samples) than the exocuticle (figure 3D), which is consistent with our previous work [35], while here we show the spatial variation as well. While apparently small, this strain at the nanofibril level is equivalent to a stress variation of approximately 180 MPa on the fibrils (stress obtained by multiplying pre-strain by axial chitin elastic modulus, taken as approx. 120 GPa from density functional theory calculations by Nikolov *et al*. [40]). This strain gradient across the cuticle is probably linked to the different levels of mineralization in the two layers known from previous work [3,17,35], as schematically shown in figure 3B, and verified in our electronic supplementary material, figure S5.

### 2.2. Distinct response of exocuticle and endocuticle to compression and tension loading

**Figure 4.**
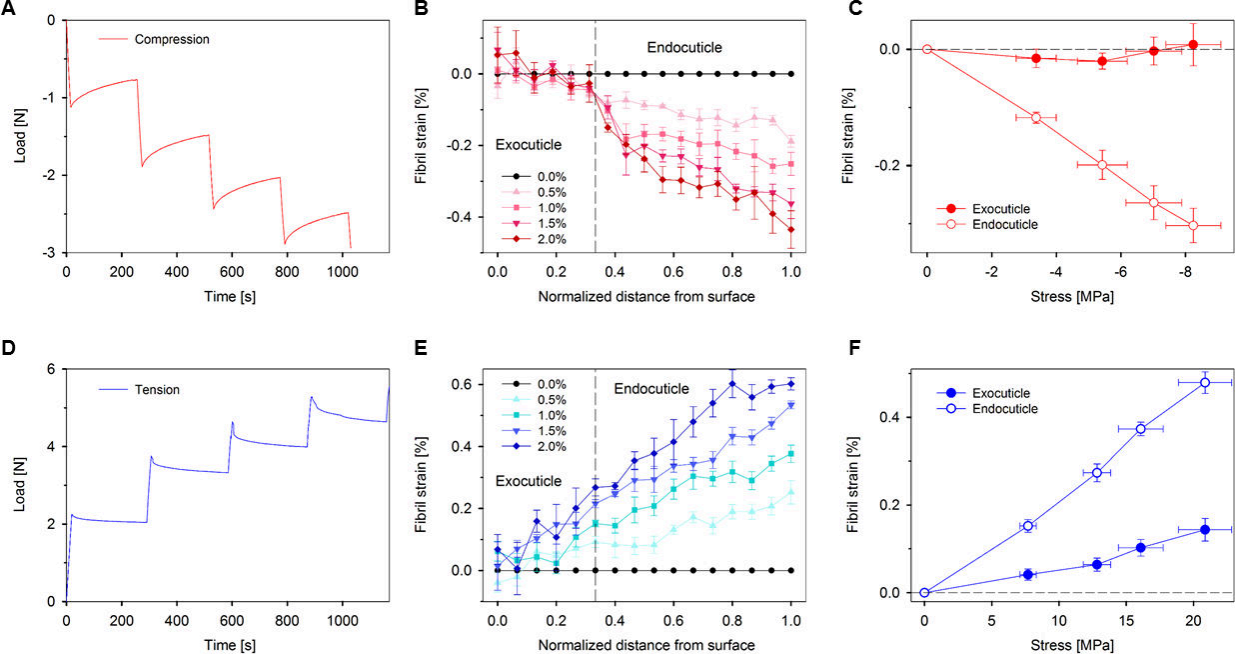
Nanoscale strain gradients in compression and tension. Chitin fibrils display a pronounced but spatially non-uniform change in fibril strain during step compression and tension. Representative applied macroscale load in stomatopod cuticle under compression (A, red) and tension (D, blue), respectively, with hold periods at 0, 0.5, 1.0, 1.5 and 2.0% of macroscopic strains. Fibril strain through the bilayer of cuticle at the strain levels of 0.0% (circle), 0.5% (triangle), 1.0% (square), 1.5% (reverse triangle) and 2.0% (diamond) under compression (B) and tension (E) loading modes. The relations of fibril strain and cuticle stress in the exocuticle (filled circles) and endocuticle (open circles) at compression (C) and tension (F). The dashed vertical lines in B and E indicate the interface of the exocuticle and endocuticle. The horizontal dashed lines in C and F show the zero-fibril strain. Data are mean ± s.e.m. (s.e.m.: standard error of mean), *n* = 4 in (B) and (C) (compression); *n* = 5 in (E) and (F) (tension).

We performed scanning X-ray diffraction with (stepwise) compression and tension experiments on the (naturally curved) abdominal tergite of the mantis shrimp; this loading mode is a simplified representation of physiological bending under impact [25]. X-ray patterns were collected on the mid-section cuticle before deformation, as well as at multiple macroscopic strain levels of 0.5, 1.0, 1.5 and 2.0%, to study how the fibrillar strains develop in the different regions at the nanoscale level (figure 4). Macroscopic cuticle stress was calculated from the load and the cross-sectional area of the cuticle sample, while the chitin fibril strain was determined by the shift in the axial lattice spacing *D*_(002)_ using the undeformed values (in a site-matched mode) as reference. Figure 4 shows—in both tension and compression—the typical macroscopic mechanical response (left), the fibril strain gradient across the cuticle and how it increases with applied load (middle) and the fibrillar strain versus cuticle stress, partitioned into exocuticle and endocuticle regions (right).

#### 2.2.1. Compression loading

In the compression mode, as the applied strain increased, the load showed a corresponding rise, reaching approximately 2.5 N when the cuticle underwent a compressive strain of approximately 2.0% at the macroscopic level (figure 4A). It is observed that at each compression level, there was a graded increase in the chitin fibril strain from the outermost exocuticle to the inner endocuticle (figure 4B; *n* = 4 samples in figure 4B,C). This gradient in fibril strain could be linked to the pre-strain and the geometry of the cuticle. In the exocuticle, the fibril strain in chitin remained relatively unchanged across different scans (figure 4B, left of dashed line), suggesting that the load transfer from the interfibrillar matrix to the chitin fibrils in the exocuticle was low, or that the fibrils existed in a pre-compressed or kinked state. Conversely, chitin in the endocuticle exhibited a tendency to bear higher deformation, with fibril strain exceeding 0.4% under the maximum compression loading.

To better display the macroscale stress in this bilayer cuticle and to compare the fibrillar response to stress across zones, we presented the averaged fibril strain of the exocuticle and endocuticle at each macroscopic strain alongside cuticle stress in figure 4C. The result emphasized that chitin fibrils in the exocuticle remained nearly non-deformed while the whole cuticle was being compressed, with fibril strains staying close to zero at all stresses, whereas endocuticle fibrils underwent significant deformation, with the compression rate (*dε*_*f*_/*dσ*_*T*_) higher than 0.037% MPa^−1^ versus approximately 0% MPa^−1^ in exocuticle.

#### 2.2.2. Tensile loading

A second loading mode (tension bending) was used to stretch out the curved cuticle and (at macroscale) mimicked the geometrical shape changes due to a localized point force at the centre of the abdominal tergite in the stomatopod, as may occur during a predatory attack in nature, or in intraspecific competition [25]. Figure 4D shows a representative example of the time-dependent stepwise tension-mode bending on the stomatopod cuticle, resulting in a load of approximately 5 N at a macroscopic tensile strain level of approximately 2.0%. Under this loading condition, chitin fibrils exhibited tensile strain at each macroscopic strain level, with fibril strain consistently increasing from the outer surface to the internal cuticle (figure 4E; *n* = 5 samples in figure 4E,F). This suggests that chitin fibrils in both exocuticle and endocuticle were subjected to tension, though with varying levels of deformation, which increased from the outer exocuticle to the inner endocuticle.

Moreover, the response in chitin fibril strain across the two layers showed a gradual change, with no significant discontinuity observed at the exocuticle and endocuticle interface. However, the increase in chitin fibril strain was notably less pronounced at all tension stages in the exocuticle, with a maximum of less than 0.3%, compared with the greater increase observed in the endocuticle, reaching around 0.6% under the maximum loading condition. The relationship between the macroscale stress and fibril strain in figure 4F reveals that chitin fibrils in exocuticle and endocuticle followed a similar response to external loading with distinct deformation rates, being significantly higher in the endocuticle (*dε*_*f*_/*dσ*_*T*_ approx. 0.023% MPa^−1^) than in the exocuticle (*dε*_*f*_/*dσ*_*T*_ approx. 0.007% MPa^−1^) under the tension loading.

To summarize the experimental findings, when the cuticle was subjected to compression or tension loading, the chitin fibrils in the endocuticle were easier to deform and were the main component to bear the stress, especially in compression where chitin in the exocuticle remained largely undeformed, while those in the endocuticle carried most of the load.

### 2.3. Matching microscale and nanoscale mechanics to a structural model

From these results, we built a model that quantifies the cuticle matrix properties, enabling the measured macro- and nano-strains. Initial estimates of cuticle moduli are from previous work [5,17]. Our model approximated the cuticle as an extensible, unshearable curved bilayer beam at the macroscale/mesoscale (layers corresponding to the exocuticle and endocuticle). Using elasticity theory [41], we derived the bilayer bending stiffness from the known displacement, stress and curvature on the cuticle. Mathematical details of our derivation are provided in the electronic supplementary material, and only the main results used to compare with the experiment are summarized here.

#### 2.3.1. Young’s moduli of the exocuticle and endocuticle

**Figure 5.**
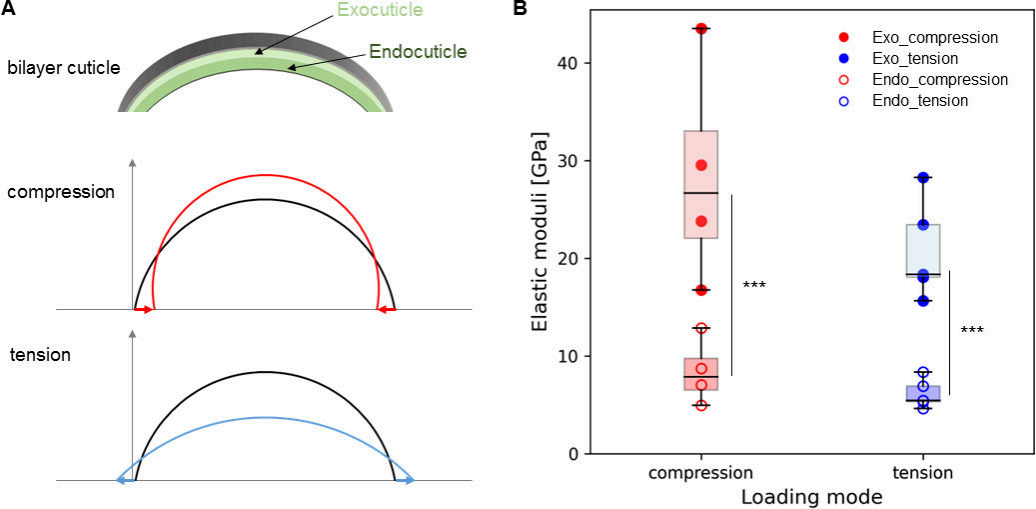
Predicted moduli from the exocuticle and endocuticle. (A) Schematic showing the bilayer cuticle consisting of exocuticle and endocuticle (top) and the cuticle arc under both compression (middle) and tension (bottom) loading modes. The arc lines (middle and bottom) indicate the undeformed (black), compressed (red) and tensioned (blue) cuticle. (B) Elastic moduli of exocuticle (red-filled circles at compression; blue-filled circles at tension) and endocuticle (red open circles at compression; blue open circles at tension). Significant differences between exocuticle and endocuticle are indicated in both loading modes; however, moduli are not significantly different between modes in each layer

From force balance in a local reference system (electronic supplementary material, figure S2), components $Q_{0}$ and $N_{0}$ ($N_{0}$ along the tangent to the deformed cuticle) are related to the (dimensionless) horizontal force ${\bar{H}}_{0}$ by


(2.1) $${\bar{H}}_{0}={\bar{N}}_{0}\cos\left(\frac{\alpha }{2}+\theta_{0}\right)-{\bar{Q}}_{0}\sin\left(\frac{\alpha }{2}+\theta_{0}\right),$$

where the values ${\bar{N}}_{0}$ (the axial force in the local reference system), ${\bar{Q}}_{0}$ (the shear force) and $\theta_{0}$ (the rotation) were obtained as outputs from differential equations solved using the shooting method described in the electronic supplementary material; α is the central angle (electronic supplementary material, figure S1). The (known) horizontal force $H_{0}$ is given by


(2.2) $$H_{0}=\;E_{1}bh\;{\bar{H}}_{0},$$

where *b* and *h* are, respectively, the width and thickness of cuticle, and ${\bar{H}}_{0}$ is calculated numerically from equation (2.1). We noted that the horizontal force $H_{0}$ does not solely depend on exocuticle modulus (*E_1_*) but also on endocuticle modulus (*E_2_*), via a ratio *η* calculated via a least-squares optimization (details in the electronic supplementary material). A sketch showing the cuticle as a bilayer curved beam with exocuticle and endocuticle labelled and the dimensions depicted is shown in electronic supplementary material, figure S3 (*t*_1_ and *t*_2_ are the thicknesses of exocuticle and endocuticle, respectively). From the above, the Young’s moduli of the exocuticle *E_1_* and endocuticle *E_2_* are obtained (figure 5). The cuticle displays significantly greater Young’s moduli in compression compared with tension (*p* = 0.009) by a factor of approximately 1.4. In both loading modes, the exocuticle has significantly higher moduli than the endocuticle (20–28 versus 6−8 GPa), consistent with prior experimental mechanical data [5,13,17]. However, we observed a discrepancy between the model and experiments (electronic supplementary material, figure S4) in tension, which suggests some mechanical yielding, or a result of the model simplification in considering the shape to be a perfect arc.

#### 2.3.2. Cuticle and fibril strain

We assumed a shearing mechanism of load transfer between the fibril and the extrafibrillar matrix at the nano- and molecular scales, as proposed in our earlier study of tensile deformation in cuticle [34] and earlier for other fibrillar collagenous biocomposites like bone, dentin and mineralized tendon [42–44]. For this, we used a modified shear-lag model to model the strain profiles at both cuticle and fibrillar levels.


(2.3) $$\epsilon_{F}\left(S,Z\right)=A_{F}\left(\kappa \right)\epsilon_{T}\left(S,Z\right),$$

where $\epsilon_{F}$ is the fibril strain, $\epsilon_{T}$ is the cuticle strain, $A_{F}(\kappa )$ is the strain concentration factor, S is the undeformed curvilinear abscissa of the bent cuticle (electronic supplementary material, figure S1) and Z is the coordinate along the cuticle thickness (electronic supplementary material, figure S3). Therefore, the strain concentration factor $A_{F}(\kappa )$ can be calculated using the equation below (from the shear-lag model in composite mechanics [45]).


(2.4) $$A_{F}\left(\kappa \right)=\frac{\epsilon_{F}\left(S,Z\right)}{\epsilon_{T}\left(S,Z\right)}=1-\frac{tanh\left(\frac{\kappa }{2}\right)}{\frac{\kappa }{2}},$$

with κ being a term depending on the cuticle matrix and geometrical parameters.


(2.5) $$\kappa =\sqrt{\frac{G_{M}}{E_{F}}}\xi_{F}\frac{1}{\sqrt{log\left(\frac{1}{\phi_{F}}\right)}}.$$

Here, $G_{M}$ is the shear modulus of the matrix, $E_{F}$ is the Young’s modulus of the fibril, $\xi_{F}$ is the aspect ratio of the fibril and $\phi_{F}$ is the volume fraction of the fibril. Here, we used literature values for $\phi_{F}$ and $\xi_{F}$ [40] along with our experimental values of κ, to get ratios for matrix modulus $G_{M}$ to fibril modulus $E_{F}$.

**Figure 6.**
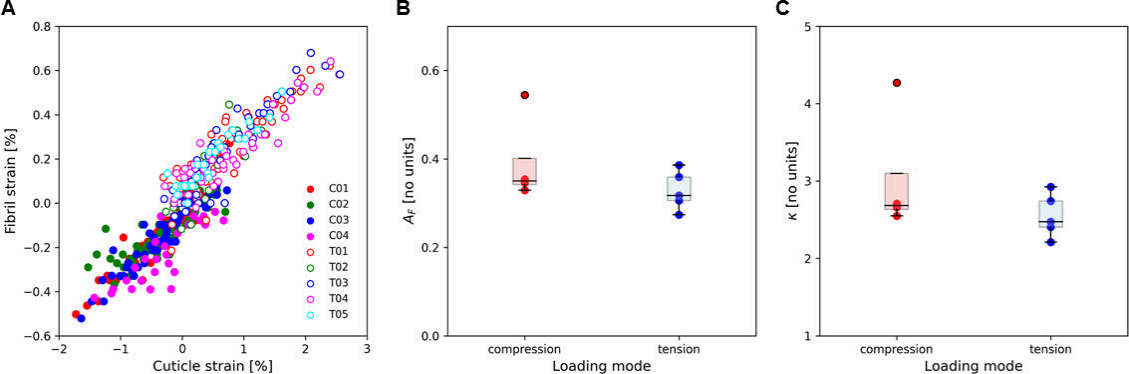
Strain concentration factors in compression and tension. (A) The cuticle strain and fibril strain for each sample at compression (filled circles) and tension (open circles). The strain concentration factor $A_{F}\left(\kappa \right)$ and κ calculated from the model at compression and tension are shown in (B) and (C), respectively. No significant differences are found between compression and tension for either parameter. The different colours in (A) indicate the individual samples. In (B) and (C), compression is shown in red and tension in blue.

According to equations (2.3)–(2.5), the strain concentration factor ($A_{F}\left(\kappa \right)$) and κ values from the strains at fibrillar (measured) and cuticle (calculated) levels across the thickness of the cuticle can be obtained as shown in figure 6. We assumed the elastic response of the cuticle to the applied loading to the first load level (which is supported by the data) led to a strain concentration factor $A_{F}(\kappa )$ of approximately 0.39 at compression (*n* = 4) and approximately 0.33 at tension (*n* = 5); the differences are not significant, possibly due to small sample numbers. The small number of samples is typical of such *in situ* scanning experiments due to the limited amount of synchrotron beamtime and the time-consuming nature of the measurements. The magnitude of the factor is consistent with our prior simpler tensile tests [6] and is perhaps linked to interfibrillar shearing, as also seen in the mineralized collagen fibres in bone (0.4−0.6 [46,47]).

## 3. Discussion

In summary, our work combines multiscale biomechanics (measuring macroscopic stresses and strains, microscale strain gradients and fibril-level strains) with modelling elastic deformation of large structures to identify how the fundamental matrix-level building blocks (chitin fibrils) of a prototypical biogenic armour (stomatopod cuticle) behave under semi-physiological flexural bending. We found an asymmetric fibrillar-level gradient in stomatopod cuticle under flexion, where the exocuticle and endocuticle deformed qualitatively and quantitatively differently in tension versus compression. This may be due to an interplay of macroscale (curved shape resulting in differing strains across the cuticle) and nanoscale (fibril pre-strain and compositional differences limiting deformability) factors (figures 3 and 4). To understand these results, we developed a modelling approach incorporating nanoscale strain measurements to predict the exocuticle and endocuticle moduli, strain concentration factor and shear-lag terms (figures 5 and 6).

While our prior work on cuticle [35] identified an intrinsic spatial gradient in the chitin molecular lattice spacing in undeformed cuticle, here we found that upon loading, these gradients are amplified (figures 3 and 4). Modulus gradients have been proposed as an evolutionary strategy to provide an optimal compromise between loading-bearing capacity and resilience (ability to recover from applied force without permanent deformation) in insect cuticles [48]. Gradients in modulus, fibre orientation and strain are a ubiquitous feature of biological composites [7], enabling, for example, controlled flexion without excessive stress concentrations. In the current study, the greater tensile pre-strain of the chitin fibrils in the endocuticle -relative to the exocuticle suggests fibrils are adapted to compressive flexion (figure 2B), where the cuticle strain in the endocuticle is negative, allowing the pre-tensed fibrils to relax. The material origins of the lowered pre-strain in the exocuticle may come from the increased mineralization, also resulting in higher elastic modulus, reduced deformability and higher resistance to impact loads. Mineralization and dehydration have been shown to reduce tensile pre-strain in other organic/inorganic biocomposites like the collagen fibrils in mineralized tissues [49]. Considering the fibrils in isolation, a static molecular-level pre-strain difference, between exocuticle (pre-compressed) and endocuticle (pre-tensed), would allow, for example, greater tensile extensibility of pre-compressed fibrils in a mineralized protein matrix up to local inelastic deformation (e.g. in the exocuticle). However, we do not observe this in the cuticle (figure 4C,F), which suggests that differences in chitin fibril/matrix interactions (e.g. due to differing levels of mineralization of the protein matrix) between exocuticle and endocuticle are playing a role. The asymmetry in the fibrillar-level strain under tensile- and compression-type flexion (figure 4C,F) may be due to (i) macroscopic sample curvature and (ii) inherent matrix differences in compression and tension, which is tested later by calculating shear-lag parameters in the model.

The observed lattice spacing change may indicate modulation of elastic energy storage ability at the fibril level, due to higher mineral content in the outer exocuticle than in the inner endocuticle (figure 3B). Pre-stress and strain build-up can occur due to dehydration in ceramics [50] and mineralization of organic scaffolds [49] and is an established strengthening strategy in e.g. pre-stressed concrete and iron girders in construction [51]. In a similar way, we speculate that alteration of pre-strain of fibrils in exocuticle and endocuticle may allow a graded impact resistance and fracture, keeping the abdominal tergite shell intact.

The integrated model/experimental approach devised here enables direct measurement of local elastic moduli and the linkage to the fibre–matrix interactions but depends on the choice of constitutive model (here a transversely isotropic elastic model was used). Use of an anisotropic model could consider the different fibril orientations in each sublamella in the plywood (Bouligand) structure of cuticle. While there is a consistently lower modulus in tension versus compression, in both exocuticle and endocuticle, the differences are not significant; this may be related to the low number of repeats in each group. On average (tension and compression), the values of the shear-lag parameter κ ~3 (figure 6) enable estimates of relative values of matrix shear modulus $G_{M}$ and fibre elastic modulus $E_{F}$, which is inaccessible to other methods. Using an aspect ratio of ξ ~ 100 and $\phi_{F}$ ~ 0.20 leads to (substituting values in equation (2.5)) $\sqrt{\frac{G_{M}}{E_{F}}}∼\frac{3log5}{100}∼\frac{1}{20}$ or $G_{M}$ ~ $E_{F}$/400. The fibre modulus $E_{F}$ is clearly much greater (400×) than the matrix modulus $G_{M}$. Previous multiscale models of the cuticle [40,52], using a different bottom-up approach to us, have incorporated a stiff matrix and stiff fibril at the nanoscale but have not reported fibril strain measurements—our work shows that fibril strains are less than the local cuticle strains, confirming that some interfibrillar shearing must be occurring. The discrepancy between our results and these previous works might be because our method might be measuring the strain of a fibril which is a composite of chitin microfibrils along with a mineralized interfibrillar matrix. By calculating $G_{M}/E_{F}$ using equation (2.5) for a range of volume fractions from 0 to 1 and aspect ratios from 1 to 20, we find that *G*_*M*_ < *E*_*F*_ for all relatively long fibres with aspect ratios greater than 5–10 and fibre volume fractions greater than 10%, which is consistent with previous structural analysis of the fibre composite structure of arthropod cuticle in lobster [22].

The difference in fibrillar strain and shear-lag parameters between the exocuticle and endocuticle might be attributed to differences in the amount of the hard mineral phase as well as the stacking density of the plywood lamella between the two layers (e.g. figure 3A, and the variable elemental distribution (from energy-dispersive spectroscopy (EDS)) of the inorganic calcium (Ca), phosphorus (P) and magnesium (Mg) between exocuticle and endocuticle in electronic supplementary material, figure S5) [3,23]. Our values for maximum tensile (approx. 20 MPa) and compressive (approx. 8 MPa) stress are consistent with some prior work (approx. 10 MPa; [53]) and lower than other values (approx. 300 MPa) on beetle cuticle [8]. This variation in mechanics between our current study and previous works may also be due to the differences in the species of animals studied, the measurement techniques utilized and the sample preparation methods, all of which have been reported to affect the biomechanics of cuticle exoskeleton [31]. Moreover, the model predicts lowered endocuticle moduli (approx. 6–8 GPa), approximately 30% of the exocuticle stiffness, in both compression and tension loading modes (figure 5B); the relative difference between exocuticle and endocuticle is consistent with microindentation or nanoindentation data across lobster and mantis shrimp cuticle [5,13,17,23,29,54]).

The modelling approach enables us to link experimental small-scale fibril-level deformation to organ-level mechanics during the deformation of the whole organ. In general, most nanoscale mechanical analyses of cuticles have used variants of scanning probe indentation to assess local mechanics. In a pioneering work on stomatopod dactyl, Weaver *et al*. [23] considered material properties only indirectly, by assigning a modulus to different regions. The work provided insight into microcrack and macrocrack nucleation but did not consider fibril-level mechanisms or the fibril/matrix separately. Considering the physiological impact on dactyl and other cuticle types, Zavattieri and collaborators [55,56] have more recently extended the analysis of such Bouligand structures and modelled and measured the effect of fibre architecture and the role of the matrix in elastic and fracture mechanics. They reported that the mechanical response of helicoidal fibrillar composites was strongly influenced by the helicoidal angle, and the helicoid architectures exhibited reduced fibre reorientation and lower strain stiffening under loading when compared with the single lamina architectures. However, their analysis did not extend to flexion or bending, and the material was a 3D-printed mimic of the biological structure (incorporating elements of nacre and cuticle) and not a real cuticle. Bottom-up elastic modelling of the cuticle by Nikolov *et al*. [40,52] (including *ab initio* modelling) incorporated structural elements like pore canals and considered variations of the relative volume fractions of chitin, protein and mineral. However, the experimental comparison was only at the macroscale and with nanoindentation data, and flexion/bending was not considered.

In comparison with these prior studies, our approach combines (i) local nanoscale resolution (of the fibril-level strain), with (ii) microscale mapping of these ultrastructural parameters across the cuticle (due to microbeam diffraction), during (iii) bending of the entire cuticle, which allows us to directly link stresses and fibril strains during semi-physiological bending and flexion modes, similar to what the cuticle may be subjected to in nature. Moreover, (iv) the modelling of large deformations in the cuticle during the experiment both accounts for the organ-level shape changes during such deformation and allows linking the external stresses to local fibril-level shear and load transfer mechanisms. While we have not taken the cuticle to macroscopic inelastic deformation here, the approach could in principle be extended to analyse the plastic or damage mechanisms in the cuticle matrix. The combined model and experimental scheme may also provide a template to integrate localized (but evolving in time) strain and stress measurements into a spatial model of mechanics.

Turning to the limitations in our work, firstly we considered, experimentally, quasi-static mechanics, rather than physiological dynamic loading of cuticle, especially in organs like the dactyl and telson [23,25]. This is necessitated by the need to perform line scans across the cuticle to visualize the strain at different macroscopic stresses. Also, our simulated extension and compression loading modes are a simplified planar representation of the type of flexion and deformation of the cuticle during predation or *in vivo* loading. However, these loading modes can be used (as in the current study) to provide the basic matrix response to load, which can be adapted to more complex organ-level deformation behaviours. This might be achieved by establishing constitutive relations linking fibril-level strain mechanisms to local stress (and e.g. mineral/matrix ratios), which could inform finite-element modelling of entire organs. Secondly, the cuticle was not kept immersed in saline during testing (which is the physiological scenario underwater) but was thawed in saline and kept in contact with wet gauze during testing. Thirdly, radiation damage from high-intensity X-ray beams may change cuticle properties as shown for other tissues like bone [57]; we mitigated this by not scanning the same location during successive line scans, but the spatial offset may introduce errors due to microstructural variations. Our mechanical data are similar between laboratory and synchrotron tests (electronic supplementary material, figure S6), showing that radiation did not significantly change the macroscale mechanics. Fourthly, the wide-angle scanning X-ray diffraction data analysis does not consider the full three-dimensional Bouligand architecture of the cuticle [18,35] but only considers the projected axial strain for the fibrils in diffraction conditions (on the Ewald sphere). Here, this is probably not a major issue since the lamellar Bouligand architecture means the cuticle is transversely isotropic when averaged across the lamellar thickness, and the elastic modulus *E* can be considered as the effective in-plane modulus of the cuticle extracellular matrix. More detailed anisotropic models considering the varying fibre orientation in the Bouligand structure could be developed, but in the experimental set-up shown here, we would only be able to measure the fibrils satisfying the Ewald condition. Fifthly, the pore–canal network running transverse to the lamellae [21] has been shown to have some load-bearing properties [35] but was not considered in the model. Sixthly, the abdominal tergite cuticle was tested in isolation from the rest of the animal; constraints due to pressure from internal tissue and from connections to adjacent exoskeletal elements will reduce total macroscopic and fibrillar-level deformability *in vivo* during bending. Lastly, since we analysed the chitin peaks from X-ray diffraction (which requires presence of Bragg peaks from a crystalline structure) combined with *in situ* loading, we cannot readily provide information on the structural deformation of the amorphous mineral/protein extrafibrillar matrix, which needs to be inferred from composite models of load transfer.

We can envisage future experiments using and extending the current approach. More complex bending and torsion loading can be conceived where lateral contraction (from equatorial reflections perpendicular to the fibril axis, shown by us previously [6]) and twisting will require the analysis of the three-dimensional diffraction model with on-axis, lateral and off-axis peaks. The Poisson ratio can be extracted from a combination of off- and on-axis measurements and modelling. Further, the impact-resistant dactyl and telson [23,25,29] were not analysed in the current work, but the same principle (sectioning followed by flexural loading) could be applied to these systems as well in the future. Incorporating tomographic techniques to model the three-dimensional structure of cuticle realistically, e.g. as done for sea urchin spines [58], to derive structure–function relations and help with interpretation of the X-ray diffraction signal, would also be an avenue for further work. To extend to dynamic loading, which is relevant for regions like the dactyl or telson subject to fast, repetitive impact forces, we could use the method described here to identify anatomical regions with distinct stress responses and focus the X-ray beam at these regions during rapid loading.

## 4. Conclusion

In summary, we developed a model to understand the experimental mechanical behaviours, at both fibrillar and whole-cuticle levels, of the stomatopod cuticle in flexion. An advantage of our method is that the structure and mechanical responses of the supramolecular building blocks of the cuticle are measured *in situ* in the natural environment rather than from a single fibril or a single molecule test. This modelling approach is not restricted to chitin-based composites and could be used in other biological and synthetic materials with gradient or hierarchical structures. It provides an experimentally validated route to extract the ultrastructural mechanisms enabling remarkable biomechanics in realistic loading environments in a range of hierarchical bioinspired and biological materials, such as horn, nacre and bone.

## 5. Material and methods

### 5.1. Cuticle preparation

Stomatopod cuticle was dissected from the abdominal tergite of frozen adult mantis shrimps (*Odontodactylus scyllarus*; figure 1A), which were obtained from a local supplier. Whole abdominal tergite cuticle was isolated using scissors and forceps and rinsed in deionized water to remove residual soft material (figure 1B). The cuticle was then sectioned into strips perpendicular to the longitudinal axis of the animal (indicated in figure 1B, around 0.7 mm wide), using a low-speed diamond blade saw (Buehler Isomet, Buehler, Duesseldorf, Germany). The curved samples with natural shape (figure 1C) were cleaned in deionized water followed by storage at −20°C (for a maximum of two weeks) for subsequent mechanical testing and WAXD measurement. To prepare the scanning electron microscopy (SEM) and EDS measurement of the cuticle cross-section, whole abdominal tergite cuticle was embedded into poly-methyl-methacrylate, sectioned into slices and then polished to a smooth finish.

### 5.2. Scanning electron microscopy measurement

The fracture surface after mechanical failure and the polished cross-section of cuticle were carbon coated and examined with a scanning electron microscope (Inspect F, FEI, Eindhoven, The Netherlands), equipped with an energy-dispersive spectrometer. EDS measurement was acquired on the polished cuticle surface at a 10 kV accelerator voltage.

### 5.3. Mechanical testing

Compression and tension were performed on the abdominal tergite samples to determine the step-level strains from the mechanics, before *in situ* WAXD measurement. The samples were hydrated after being taken out from the freezer, followed by dimension measurement using callipers. The cuticle specimens were then fixed between the grips with both ends clamped with sandpaper in a custom-made uniaxial micromechanical tester, developed for biological systems [6], with a 110 N load cell (RDP Electronics, UK) and a DC motor (M126.DG, Physik Instrumente, UK; figure 1G). A LabVIEW control interface (National Instruments, UK) was used to record the load and displacement data. A 0.1 N tare load was initially applied to the samples before compression/tension loading at a strain rate of 0.05% s^−1^. The overall displacement of 0.1 mm was determined to be used in the *in situ* mechanical testing, where the samples were deformed safely as well as not fractured yet when loaded with a gauge length of approximately 5 mm.

### 5.4. *In situ* wide-angle X-ray diffraction measurement

A stepwise compression/tension was applied on the cuticle during synchrotron X-ray measurements, as shown in figure 2B. X-ray patterns were first taken in the middle of the samples across the cuticle before any loading was applied. The samples were then compressed/tensioned to successive macroscopic strains of 0.5, 1.0, 1.5 and 2.0%, holding for a stress relaxation period, and line scans were conducted across the sample when the load was approaching constant.

WAXD experiments were carried out on the microfocus end-station at beamline I22 at Diamond Light Source (DLS, Harwell Campus, UK) [59]. The micromechanical tester was mounted on the platform to allow simultaneous mechanical testing of the samples as well as any tester movement during WAXD measurements. The synchrotron X-ray beam size was 15 µm at an energy of 14 keV. The X-ray beam diameter is much larger than expected fibril diameter of approximately 100–200 nm, and thus the X-ray diffraction patterns report the averaged molecular response to stress—at the nanoscale—of many fibrils within the microscale scattering volume. A Pilatus P3-2M detector was used to record WAXD patterns with a pixel resolution of 1475 × 1679 pixels and a size of 172 µm. The sample-to-detector distance was calibrated using silver behenate.

WAXD patterns were collected across the bilayer at the middle of the cuticle at each step strain. For the line scans, the micro-tester stage was shifted 20 µm in the horizontal direction with respect to the X-ray beam after each point to obtain the patterns from both exo-layer and endo-layer (figure 2C). Between each strain level applied, a vertical stage shift of 25 μm was used prior to the next WAXD line scan, so that the exposed ranges for line scan at each strain were close but not the exact same regions to minimize radiation damage to the cuticle. At the length scale of approximately 100 μm (corresponding to the typical five line scans/sample), the thickness of the abdominal tergite cuticle is nearly constant (at larger length scales the abdominal tergite curvature will be noticeable). Further, all line scans were re-aligned such that the first diffraction pattern containing signal from the cuticle is from the endocuticle. Thus line scans can be aligned for each sample to ensure comparability. Across different samples, the varying thickness was normalized. The exposure time was 1 s per measurement point, while the time interval between WAXD acquisitions was 0.02 s.

### 5.5. Wide-angle X-ray diffraction data analysis

Diffraction peak (002), of which *q* is approximately 12.15–12.25 nm^−1^ and oriented along the chitin fibril direction, was used to study the feature of chitin fibrils and the loading-induced deformation at the nanoscale. A cake-shaped sector of the diffraction peak (002) (figure 2E) was integrated azimuthally for each WAXD frame to get the intensity profile *I*_(*q*)_, which was then fitted to a Gaussian with a linear background to obtain the peak position *q*_(002)_ and fibrillar spacing *D*_(002)_, where *D*_(002)_ is equal to 2*π*/*q*_(002)_ (figure 2F). The shift in the peak position *q*_(002)_ was used to calculate fibril strain, as described previously [6]. The reference *q*_(002)_ or *D*_(002)_ used to determine the fibril strain was taken as the value in the undeformed state before loading. FIT2D software [60] and Python package *lmfit* [61] were used to perform the integration and fitting for the WAXD data.

The location of the samples after each strain applied as well as the interface of exocuticle and endocuticle were determined by the intensities of the WAXD diffractions in the integration region of approximately 11.85–19.05 nm^−1^. From the intensity mapping (figure 2C), the position and the loading-induced shift of the samples could be observed as well as the exocuticle and endocuticle distinguished. Note that due to the diffraction geometry, the (002) peak reflects only the contributions from fibrils (close to) in-plane perpendicular to the X-ray beam and not those of fibrils at large out-of-plane angles, i.e. near parallel to the X-ray beam. When displaying the change in *D*_(002)_ and the fibril strain (figures 3C and 4B,E) across the cuticle, the positions where the data were taken were normalized to the thickness of each cuticle sample, taking the measurement point at the exocuticle surface as zero to minimize the thickness variation between samples.

## 6. Statistical analysis

The representative traces refer to individual samples, and the grouped data are mean values with standard error (s.e.m., sample number *n* = 4 (compression), 5 (tension) and 9 (total)). The plots were made in SigmaPlot (v. 12, Systat Software, Inc.) and Python (Anaconda Software Distribution, Anaconda Inc.). Statistical analysis was carried out in R (v. 4.4.2, https://www.r-project.org/) using RStudio. Data normality was assessed using the Shapiro–Wilk test. The change in the lattice spacing D_(002)_ of chitin in the cuticle’s initial state (before loading) and the elastic moduli within and between different loading mode groups showed normal distribution, while the strain concentration factors were not normally distributed. Therefore, considering also the small sample numbers involved, non-parametric statistical tests were performed. Specifically, to compare the lattice spacing (figure 3D; before loading) between the exocuticle and endocuticle, all samples were pooled (*n* = 9) and the Wilcoxon signed-rank test (with paired = TRUE) was used. For the comparison of elastic moduli during loading (figure 5B), the non-parametric aligned rank transform test with a mixed (split-plot) design was used to test for main effects (loading mode and exocuticle/endocuticle) and interaction, followed by pairwise *post hoc* tests comparing the moduli between exocuticle and endocuticle in each loading mode. Finally, the strain concentration factors $A_{F}\left(\kappa \right)$ and κ were compared between compression and tension using the Mann–Whitney test with Holm adjustment for multiple comparisons (as $A_{F}\left(\kappa \right)$ and κ are related measures). The statistical significance was set at *p* < 0.05 and indicated as *p* < 0.05 (*), *p* < 0.01 (**) and *p* < 0.001 (***).

## Data Availability

The Matlab code and data used in the paper are available on Zenodo at: [62]. Supplementary material is available online [63].
